# Who Watches Live Streaming in China? Examining Viewers’ Behaviors, Personality Traits, and Motivations

**DOI:** 10.3389/fpsyg.2020.01607

**Published:** 2020-08-04

**Authors:** Yi Xu, Yixin Ye

**Affiliations:** ^1^USC-SJTU Institue of Cultural and Creative Industry, Shanghai Jiao Tong University, Shanghai, China; ^2^Department of Psychology, School of Social Development and Public Policy, Fudan University, Shanghai, China

**Keywords:** live streaming, personality traits, motivations, social media, online tipping

## Abstract

With millions of viewers globally, live streaming is a new social media that can deliver video content in real time and with many social interaction functions. Our research aims to understand the personality traits and the motivations of active live streaming viewers as well as their user behaviors in the general population in China. Our results indicate that extraversion was negatively associated with live streaming use, while openness was positively associated. The main motivations to watch live streaming were social interaction, information gathering, and entertainment, and they were associated with different frequencies of use and genre selection. Financial tipping behavior was positively associated with social interaction. Furthermore, motivations mediated the effects of personality traits on live streaming use. People high in openness were more likely to be motivated to chat by information needs. Among extraverts, those who were more social watched fewer streams. We demonstrated that personality traits and motivations can jointly predict live streaming use. The current study not only provides the first evidence of live streaming use with personality traits and motivations but also expands the perspective on individual difference with the mediation analysis. Practically, the person–situation joint interpretation can give industry a clear indication on how to design personalized user experience for people with different personality traits and motivations.

## Introduction

Live streaming—a new way to deliver video content in real time—has attracted millions of users globally in recent years. The popularity of digital cameras and the increased availability of network access have facilitated the substantial growth of video transmission on the Internet. In 2016, live streaming ranked as the top application of mobile data traffic and accounted for over 34% of total mobile data (Informa Telecoms and Media, 2016). Since 2015, popular services such as YouTube, Facebook, and Twitter have all launched live streaming functions. Twitch, a popular live streaming platform owned by Amazon, boasts over 188 million monthly viewers and 5.5 million monthly broadcasters (Twitch tracker, 2020). In China alone, in 2020, there were more than 559 million live streaming users across about 270 platforms, implying 62% penetration of China’s Internet users (CNNIC, 2020). Who are the users of live streaming in China? Why do they adopt this new media application? The current research seeks to answer the questions by examining the relationship between psychological individual differences of viewers and their behavior engagement.

From a user’s perspective, we could draw more inferences about how personality traits and motivations can influence live streaming use. Furthermore, our study of live streaming use in China can contribute to the current body of social medial research from a cultural perspective, providing insights of how culture may influence the way people use live streaming. In addition, our study can offer practical implications for platforms to attract users based on individual needs and personality traits.

## Live streaming

Social live streaming services (SLSSs) belong to the broad category of social network sites (SNSs), while featuring specific characteristics: synchrony, real-time broadcasting of user-generated content, interactions between the viewers and the streamers, and a gratification system (Scheibe et al., 2016). Through live streaming, ordinary people can create content relevant to their own interests and reach niche viewers who share those interests (Lu et al., 2019a). Users are not only consumers and data providers but also content producers as well as volunteers or aspiring professionals in the emerging labor market (Van Dijck, 2009). This creates a diversity of streamers and contents of live streaming, such as game, sport, news, and performance and celebrity shows and a “closer” relationship between streamers and viewers. The interaction between the streamers and the viewers is two-way. During a broadcast, streamers are in the focal point. They can directly acknowledge and respond verbally to viewers, while viewers often type in comments. Viewers can influence the broadcasts by sending virtual gifts to support streamers. Meanwhile, viewers can communicate with each other *via* comments or emojis. Therefore, often there is an interesting cross-model discourse during online streaming (Recktenwald, 2017). As such, the interactions—between the viewers and between the streamers and the viewer—provide a much lively social interaction experience.

## Literature Review and Hypothesis Development

A few studies have examined live streaming in the United States, mainly focusing on the Twitch platform (Sjöblom and Hamari, 2017). For example, Woodcock and Johnson (2019) examined the character action of Twitch streamers, including being friendly to viewers, soliciting donations, building parasocial intimacy with spectators, and engaging audiences through humor. Hilvert-Bruce et al. (2018) explained Twitch live streaming viewer engagement from four aspects: emotional connectedness, time spent, time subscribed, and donations. Despite the fact that China has the largest and fastest-growing group of live streaming users (CNNIC, 2020), very few studies have examined Chinese users. Among a few notable exceptions, some researchers have examined live streaming from the perspective of streamers using a case study of female streamers (Zhang and Hjorth, 2017). Zou (2018) discussed informational capitalism by looking into the structure and affordances of live streaming platforms. In terms of live streaming behaviors, Zhou (2017) explored the associations among users’ demographics, usage, and perceptions of live streaming. Lu et al. (2018) investigated how content influences viewing behavior and engagement in Chinese live streaming.

However, research from individual perspectives concerning the factors leading to the use of live streaming in China has been limited. A growing body of evidence suggests that personality traits and motivations are influential in guiding online behavior [for personality traits, e.g., Ross et al. (2009); for motivations, e.g., Jung et al. (2007)]. Therefore, the present study aims to examine individual differences in live streaming use in China.

In the current study, we focus on the viewers, who are the majority of live streaming users. We define live streaming use as viewing streams and communicating through chatting, which are the major basic functions on the platforms. We are also interested in the virtual gifting behavior. As a voluntary payment behavior, it is less commonly used for only around 40% of viewers indicated that they have spent money on paid virtual gifts (iResearch, 2017). Thus, we structure our research questions as follows:

Research question 1: What personality traits of the viewers are associated with live streaming use?
Research question 2: What are the motivations in live streaming use and how do these motivations influence user behavior? In particular, what motivations are associated with virtual gifting behavior?

### Personality Traits and Live Streaming Use

Personality traits as relatively stable descriptors of individuals’ behavior have been used to characterize individual differences since 1950 [for a review, see Schultz and Schultz (2005)]. The “Big Five” personality traits—extraversion, neuroticism, openness to experience, agreeableness, and conscientiousness (Costa and McCrae, 1988)—have been widely used to understand how personality contributes to an individual’s behavior.

Briefly, extraversion focuses on sociability, reflecting the tendency to be with others and seek social simulation. Extraverts are typically adventurous, sociable, and talkative. Neuroticism focuses on the experience of negative emotions, such as depression, pessimism, and feeling vulnerable. Openness to experience refers to being creative and open to change. People high in openness normally have broad interests and seek out new and novel experiences. Conscientiousness refers to planning, organization, and perseverance. Conscientious people are dutiful and responsible in their tasks. Agreeableness is about trust, honesty, compliance, and friendliness.

Cheng et al. (2019) found that agreeableness was negatively and neuroticism was positively related to the addictive use of live video streaming, but other personality traits showed no significant relationship. Other scholars looked into the personality patterns of streamers and suggested that low in openness, conscientiousness, and extraversion but high in neuroticism during the streaming tend to own more popularity (Zhao et al., 2019). However, to our knowledge, no study has yet examined how personality traits are associated with the viewers’ general live streaming use. Thus, we grounded our hypotheses in broad SNS research and considered the features of live streaming.

Previous studies indicate that extraverts are sociable and talkative people actively engaging in various activities in the virtual world. For example, extraverts were found to belong to more Facebook groups (Ross et al., 2009) and were more likely to use the communicative functions of SNS (Wang et al., 2012). However, the negative association of extraversion and SNS use was supported by findings from Wang et al. (2012) in using the online gaming functions of SNS. Mark and Ganzach (2014) explained that the popularity of SNS may contribute to the conflicting results. When the popularity grows, more people switch to those online social networking platforms. Therefore, earlier studies tended to find a negative relationship, whereas later findings tended to observe a positive relationship [see Amiel and Sargent (2004) and Mark and Ganzach (2014)]. The level of popularity may also explain the preference of functions of SNSs in the study of Wang et al. (2012). In addition, unlike streamers who are on the stage, viewers in live streaming are often confined in texts, which may also discourage extravert people. Given that live streaming has gained popularity in recent years (Long and Tefertiller, 2020) as well as the viewer’s role in live streaming platform, we propose:

H1: Extraversion is negatively associated with viewers’ live streaming use.

The neuroticism–loneliness hypothesis proposed that neurotics could use the Internet to avoid loneliness and escape from everyday life (e.g., Hamburger and Ben-Artzi, 2003). Findings have supported that neurotics demonstrate a strong interest in using Facebook for socializing (Ryan and Xenos, 2011; Hughes et al., 2012). The room setting in live streaming forms groups of people with similar interests and facilitates group communication with real-time chat functions. Thus, we suggest this association stands in live streaming:

H2: Neuroticism is positively associated with viewers’ live streaming use.

Individuals high in openness are curious and looking for change and novelty (McCrea and Costa, 1999). This relates to seeking novel experiences in SNS with various functions (e.g., Correa et al., 2010; Wang et al., 2012; Mark and Ganzach, 2014). Nevertheless, contrary findings have suggested that openness is not associated with SNS use (Wilson et al., 2010; Hughes et al., 2012) and is even negatively associated with posting selfies on SNSs (Choi et al., 2017), which could be related to the characteristics of different SNS applications. We believe that the tendency to seek novel experiences could be associated with trying new applications of live streaming as well as the rich and diverse content on the platform, and we hypothesize that:

H3: Openness is positively associated with viewers’ live streaming use.

From the literature, agreeableness and conscientiousness are comparably less clearly related to social media use (e.g., Ross et al., 2009; Hughes et al., 2012). For example, agreeableness is not related to online contact (Ross et al., 2009), online communication (Mark and Ganzach, 2014), or number of friends on SNSs (Wang et al., 2012). Agreeableness is about trust, warmth, and honesty, which does not suggest particular engagement with live streaming use. Conscientiousness is characterized by achievement-driven and planned behavior, which is often associated with educational achievement (Barrick and Mount, 1991). Live streaming provides a wide variety of leisure content as well as educational programs, which may make the relationship between live streaming use and conscientiousness difficult to identify. Therefore, we suggest:

H4: Agreeableness is not associated with viewers’ live streaming use.
H5: Conscientiousness is not associated with viewers’ live streaming use.

### Motivations and Live Streaming Use

Gros et al. (2017) identified three motivations for using Twitch, including entertainment, information seeking, and socialization. A recent study also suggested that the three most important motivations of using Facebook live are entertainment, sharing opinions and experience, and socialization (Skjuve and Brandtzaeg, 2020). The content of live streaming such as games, performance, and celebrity shows can provide great entertaining experience. Chen and Lin (2018) surveyed Taiwanese people who watch live streaming *via* social network sites and demonstrated that entertainment can drive the usage. Regarding information seeking, a study examined user’s information behavior by analyzing the chat logs on Twitch and suggested that topics evolved constantly into important sources of information (Diwanji et al., 2020). In live streaming, viewers can watch not only news but also educational streams about various topics. Lu et al. (2019a) examined the knowledge-sharing streams in China and found that many viewers were motivated to learn from Intangible Cultural Heritage masters through live streams. As social interaction is emphasized in SLSSs use, many studies have documented social interaction as a major motivation for live streaming use (Hu et al., 2017; Wohn et al., 2018). For example, social interaction motivates people to watch different content genres from game streams (Sjöblom and Hamari, 2017) to outdoor and real-life streams (Lu et al., 2019b). Hilvert-Bruce et al. (2018) found that, on Twitch, viewers who preferred small channels were more motivated by social engagement than those who preferred larger channels. Therefore, we propose the hypotheses that:

H6a: Entertainment is one of the motivations of live streaming users and is positively associated with live streaming use.
H6b: Information seeking is one of the motivations of live streaming users and is positively associated with live streaming use.
H6c: Social interaction is one of the motivations of live streaming users and is positively associated with live streaming use.

In addition, Winter et al. (1998) proposed that motivation can mediate the effects of personality traits to predict behavior. From a social cognitive perspective, personality-trait-related differences could appear as differences in perceived efficacy (Bandura, 1977), which could be then associated with motivations. Extensive evidence supporting that self-efficacy belief can affect motivation has been accumulated. For example, writing self-efficacy belief is significantly associated with writing motivation (e.g., Pajares, 2003). Therefore, people’s belief in their personal efficacy to interact with others can determine their levels of social motivation. Hence, extraversion facilitates social motivation, whereas introversion deflects it.

In addition, Palmgreen and Rayburn (1982) proposed an expectancy value model which can be used to explain the relationship between personality traits and motivations in media use. They claimed that gratifications from media are a function of a person’s beliefs that the media possess certain attributes mediated by the subjective evaluations of these attributes. In the case of live streaming, for extraverts who value socialization, their gratifications could be associated with how the platforms can fulfill their social needs. According to the neuroticism–loneliness hypothesis, neurotics have a strong need of socialization. Similarly, people high in openness who look for diversified content in live streaming could be associated with information motivation. Therefore, we hypothesize that:

H7a: Social motivation can mediate the effects of extraversion in live streaming use.
H7b: Social motivation can mediate the effects of neuroticism in live streaming use.
H7c: Information motivation can mediate the effects of openness in live streaming use.

Nevertheless, several studies found multiple mediation relationships between personality traits and motivations. For example, Graham and Gosling (2013) found that individuals motivated by social networking to play *World of Warcraft* were high in extraversion, agreeableness, neuroticism, and openness. Johnson and Gardner (2010) found that the more open to experience people are, the more likely they are to be motivated by entertainment to play video games. We cannot exclude the possibility of other mediation patterns. Therefore, we would also explore other potential mediation patterns.

While advertising and subscription are major revenue sources for live streaming platforms in western countries, platforms in China mostly monetize by “tipping” system (Li et al., 2018). On Chinese live streaming platforms, users can spend real money to buy virtual gifts and send to streamers during a broadcast. Streamers can cash out 30–65% of the amount of the virtual gifts, and the platforms take the rest, thus providing an important source of income for the streamers as well as the platforms.

Recently, research investigated what drives people to tip and found that social interaction plays an important role (Deng and Chau, 2019; Lee et al., 2019). Lu et al. (2018) interviewed users and suggested that tipping facilitates users to present and express themselves. Tipping also helps viewers win attention and admiration from other viewers. Guan et al. (2020) proposed a model and argued that tipping can build up swift *guanxi* between streamers and viewers. This is consistent with previous findings of virtual goods in online games. The findings suggested that the sociability of online games is significantly associated with the intention to purchase (Animesh et al., 2011) and how much real money players spend to purchase virtual goods (Lee and Wohn, 2012). Therefore, we hypothesize that:

H8: Social interaction is positively associated with tipping behavior in live streaming.

## Materials and Methods

### Participants

An online survey was conducted with 332 adults (18 years old or older) *via* a Chinese survey platform^1^. The platform has more than two million users nationwide. In our analysis, we included only “active viewers” who reported watching live streaming at least 1 day per week, without specifying the platforms that they use. Thus, the final dataset included 210 participants (50% of the participants were males, and 82.9% were between 26 and 40 years old). These participants were from 24 of the 31 provinces in China, including major metropolitan areas and highly populated provinces such as Shanghai (15.7%), Beijing (11.0%), Guangdong (13.8%), Shandong (8.1%), Jiangsu (6.7%), and other provinces.

### Measures

#### Viewers’ Behaviors

As described above, viewers can watch and chat during live streaming. Therefore, we used frequency of watching and frequency of chatting to describe viewers’ behaviors. The participants were asked how many days per week they watch live streaming (labeled as days, 1 = less than 1 day, 2 = 1 or 2 days, 3 = 3 or 4 days, 4 = 5 or 6 days, and 5 = 7 days), how many streams they watch per day^2^ (labeled as streams, 1 = less than one stream, 2 = one or two streams, 3 = three or four streams, 4 = five or six streams, and 5 = more than six streams), and how many times they send messages while watching a live stream (labeled as chat, 1 = never, 2 = one or two times, 3 = three to five times, 4 = more than five times, and 5 = active chatting). *Days* was used to capture whether watching live streaming was a daily activity, while *streams* measured the involvement based on the counts of the live streams. Viewers who use live streaming more often can be indicated by either frequency of watching (days and streams) or frequency of chatting (chat). Additionally, the participants were asked to indicate the genres of live streams that they usually watched from the options *education* (providing knowledge-based content and training courses on various topics), *news* (news from TV channel official account, editorial news, and spot news), *sports* (live sports events and sports game commentary), *games* (showing the play of video games), *performance* (singing, dancing, or other performance by amateurs), *celebrity* (hosted by famous singers, actors, or other celebrities), and *life* (such as make-up, social eating, feeding pets, creative process of making things or projects, and miscellaneous topics). The categories were adapted from an industry report^3^ (iResearch, 2016). For tipping behavior, we measured the actual behavior by asking the participants whether they had spent money to send virtual gifts to streamers (labeled as *spend*).

#### Personality Traits

Personality traits were measured by the Ten-Item Personality Inventory in China (TIPI-C) (Li, 2013). This scale was devised as a brief measure of The Big Five dimensions of personality for Chinese participants. The TIPI-C has adequate levels of validity and reliability (for subscales, alpha > 0.60). The participants were asked to rate their agreement with different pairs of traits (seven-point Likert scale, from 1 = not at all like me to 7 = very much like me).

#### Motivations

We used a 15-item scale to assess why people watch live streaming. Items that described motivations of social interaction, entertainment, and information seeking were adapted from previous studies that examine motivations to use SNSs and play social network games (Kim et al., 2011; Lee and Wohn, 2012). Then, we recruited a small group of live streaming viewers, which are around 20 active users, to provide their ideas on the motivations of live streaming use *via* online chat (see Appendix 1). Based on the interview, some items were adjusted to fit in the live streaming context (e.g., “to support talented streamers”). The participants were asked to rate their agreement with different motivations for watching live streaming (seven-point Likert scale, from 1 = *not at all like me to 7* = *very much like me*). The study sample was considered adequate (KMO = 0.82). The results of a principal component factor analysis revealed three factors: social (Cronbach’s α = 0.87), entertainment (α = 0.68), and information (α = 0.57), which explained 61.1% of the variance.

## Results

### Descriptive Results

Table 1 presents the descriptive statistics of the study sample. A majority of the sample was married (77.6%), had a bachelor’s degree or above (86.6%), and had a monthly income above 5 K RMB (approximately 734 USD, 82.4%). To understand whether our sample was representative of Chinese live streaming viewers in general, we compared the demographics of our participants with an industry report and found that the characteristics of our sample are similar to those in the report (iResearch, 2016).

**TABLE 1:** Descriptive statistics of the study sample.

	**% (*N* = 210)**
**Age (in years)**	
18–25	7.10
26–30	38.10
31–40	44.80
41–50	6.70
51–60	2.90
>60	0.50
**Income (monthly, RMB)**	
<3 K	3.80
3 K–5 K	13.80
5 K–10 K	61.00
10 K–20 K	19.00
20 K–50 K	2.40
**Education**	
Elementary	0.50
Technical secondary	0.50
High school graduate	1.90
Technical college	10.50
College graduate	77.10
Master’s degree	8.10
PhD or above	1.40
Male	50.00
Married	77.10

Most of the active viewers (74.3%) watched live streaming more than 3 days per week: 25.7% watched on only 1 or 2 days, 38.1% watched on 3 or 4 days, 23.3% watched on 5 or 6 days, and 12.9% watched every day per week. Regarding streams, 12.9% users did not finish one stream per day, 63.3% watched one or two streams per day, and 23.8% were heavy users who watched three or four streams per day. Most of the users enjoyed chatting during live streaming: 92.8% of users chatted at least once per stream, including 38.1% who chatted three to five times, 8.1% who chatted more than five times, and 7.1% who were active/frequent chatters. Moreover, 64.3% of the participants had spent money to send virtual gifts to streamers during live streaming.

As for the genres of live streams, life (61.0%), celebrity (54.8%), and games (48.6%) were most popular; other interests included sports (39.0%), news (38.6%), performance (38.1%), and education (29.0%). Most people (95.7%) chose more than one genre they liked. The participants were motivated to watch live streaming by entertainment (*M* = 5.54, SD = 0.71), information (*M* = 5.18, SD = 1.16), and social (*M* = 4.27, SD = 1.13). As for personality traits, our participants were high in conscientiousness (*M* = 5.15, SD = 1.03), agreeableness (*M* = 4.96, SD = 0.94), openness (*M* = 4.88, SD = 1.10), and extraversion (*M* = 4.86, SD = 1.25). However, they were comparably low in neuroticism (*M* = 3.01, SD = 0.93).

We found that those who watched streams more often also chat more often (see Appendix 2, zero-order correlational matrix). There were significant correlations between days and chat (*r* = 0.43, *p* < 0.01) and between streams and chat (*r* = 0.23, *p* < 0.01). It seems that married people (*r*_chat_ = 0.24, *p* < 0.01), people with higher incomes (*r*_days_ = 0.17, *p* < 0.01; *r*_streams_ = 0.21, *p* < 0.01; *r*_chat_ = 0.17, *p* < 0.05), and those with higher education (*r*_days_ = 0.17, *p* < 0.05; *r*_s__treams_ = 0.16, *p* < 0.05) were more active viewers (Table 2). However, gender was not associated with the frequency of live streaming use nor with motivation.

**TABLE 2:** Correlational analysis of user behaviors and demographic variables.

**Variables**	**1**	**2**	**3**	**4**	**5**	**6**	**7**
1. Days							
2. Streams	0.26**						
3. Chat	0.43**	0.23**					
4. Gender^a^	–0.04	–0.07	0.06				
5. Age	–0.03	0.01	–0.01	−0.15*			
6. Income	0.17*	0.21**	0.17*	0.06	0.15*		
7. Education	0.17*	0.16*	0.07	0.03	–0.08	0.29**	
8. Marital status^b^	0.06	0.1	0.24**	0.03	0.44*	0.23**	0.10

^*a*^1, male; 2, female. ^*b*^1, single; 2, married. *N* = 210; * *p* < 0.05, ** *p* < 0.01.

Chi-square test was used to explore the relation between genres of live streams and demographic variables. There were significant gender differences in the preferred genres. Females watched education (χ^2^ = 5.20, *p* < 0.05), celebrity (χ^2^ = 8.48, *p* < 0.01), and life (χ^2^ = 15.69, *p* < 0.01) more than males did, while males preferred sports (χ^2^ = 23.13, *p* < 0.01) and games (χ^2^ = 12.89, *p* < 0.01). News and performance were watched more often by married than single participants (χ^2^_news_ = 14.33, *p* < 0.01; χ^2^_performance_ = 4.05, *p* < 0.05) and elder than younger adults (χ^2^_news_ = 13.60, *p* < 0.05; χ^2^_performance_ = 12.01, *p* < 0.05).

### Personality Traits and Viewers’ Live Streaming Use

We conducted hierarchical regressions using days, streams, and chat as dependent variables and personality traits as independent variables (Table 3). After the demographic variables were controlled for, personality traits explained an additional 3–6% of the variance of the regression models. Extraversion was associated with less live streaming use (β_streams_ = −0.19, *p* < 0.05). Openness was a significant predictor of days and chat (β_days_ = 0.23, *p* < 0.05; β_chat_ = 0.29, *p* < 0.01). We also used logistic regression to explore the relations between personality traits and genres of streams. People high in openness chose more games (OR = 1.83, *p* < 0.01), while more agreeable people had less interest in news (OR = 0.65, *p* < 0.05).

**TABLE 3:** Hierarchical regressions examining viewers’ behaviors with personality traits.

	**Model 1**			**Model 2**		
	**Days**	**Streams**	**Chat**	**Days**	**Streams**	**Chat**
Gender^a^	–0.07	–0.10	0.02	–0.07	–0.10	0.03
Age	–0.08	–0.06	–0.15	–0.05	–0.04	–0.12
Income	0.14	0.17*	0.13	0.12	0.17*	0.12
Education	0.11	0.11	–0.02	0.11	0.11	–0.03
Marital status^b^	–0.05	–0.08	−0.27**	–0.03	–0.08	−0.24**
Openness				0.23*	0.14	0.29**
Conscientiousness				–0.10	0.04	0.00
Extraversion				–0.03	−0.19*	–0.05
Agreeableness				–0.07	–0.04	–0.10
Neuroticism				0.01	0.04	0.04
Adjusted *R*^2^	0.03	0.04	0.07	0.05	0.05	0.11
△*R*^2^				0.04	0.03	0.06*

The numbers show standardized beta coefficients. ^*a*^1, male; 2, female. ^*b*^1, single; 2, married. *N* = 210; * *p* < 0.05, ** *p* < 0.01.

Thus, we found supporting evidence for H1 that extraversion is negatively associated with viewers’ live streaming watching, for H3 that openness is positively associated with live streaming use in terms of both watching and chatting, and for H5 that the effects of conscientiousness were insignificant. H2, which hypothesized that neuroticism would be positively associated with live streaming use, was not supported. For H4, we only found agreeableness to be associated with one genre preference.

### Motivations and Viewers’ Live Streaming Use

The regression analysis indicated that social (β_days_ = 0.21, *p* < 0.05) and information (β_days_ = 0.21, *p* < 0.05; β_chat_ = 0.21, *p* < 0.01) were significant predictors in live streaming use (Table 4). After the demographic variables were controlled for, motivations explained an additional 4–8% of the variance of the regression models. Therefore, these results support H6b and H6c.

**TABLE 4:** Hierarchical regressions examining viewers’ behaviors with motivations.

	**Model 1**			**Model 2**		
	**Days**	**Streams**	**Chat**	**Days**	**Streams**	**Chat**
Gender^a^	–0.07	–0.10	0.02	–0.08	–0.08	0.01
Age	–0.08	–0.06	–0.15	–0.08	–0.03	–0.14
Income	0.14	0.17*	0.13	0.13	0.15*	0.11
Education	0.11	0.11	–0.02	0.10	0.10	–0.04
Marital status^b^	0.05	0.08	0.27**	0.00	0.05	0.21**
Social				0.08	0.20*	0.12
Entertainment				0.05	–0.12	0.00
Information				0.19*	–0.02	0.21**
Adjusted *R*^2^	0.03	0.04	0.07	0.08	0.07	0.13
△*R*^2^				0.06**	0.04*	0.08**

The numbers show standardized beta coefficients. ^*a*^1, male; 2, female. ^*b*^1, single; 2, married. *N* = 210; * *p* < 0.05, ** *p* < 0.01.

The logistic analysis revealed that people motivated by the social factor preferred games (OR = 1.54, *p* < 0.05), performance (OR = 1.56, *p* < 0.05), and celebrity streams (OR = 1.63, *p* < 0.01) (Table 5). People who looked for entertainment were likely to watch fewer education streams (OR = 0.50, *p* < 0.01) but more games (OR = 1.61, *p* < 0.05) and performance streams (OR = 1.60, *p* < 0.05). Those who wanted to obtain information watched more sports (OR = 1.56, *p* < 0.01) and life streams (OR = 1.47, *p* < 0.05) but fewer performance streams (OR = 0.67, *p* < 0.05).

**TABLE 5:** Logistic regressions (LR) predicting genres of streams with motivations.

	**Education**	**News**	**Sports**	**Games**	**Performance**	**Celebrity**	**Life**
Gender^*a*^	2.13*	0.82	0.21**	0.31**	0.73	2.41**	3.27**
Age	1.02	1.43	1.19	0.95	0.87	0.77	1.19
Income	1.26	0.63*	1.02	1.31	0.97	1.44	0.85
Education	1.17	1.52	1.53	0.93	1.11	0.81	0.96
Marital status^*b*^	0.96	3.52*	0.45	0.97	3.10*	0.97	0.70
Social	1.31	1.06	0.89	1.54*	1.56*	1.63**	0.76
Entertainment	0.50**	0.75	0.91	1.60*	1.61*	0.78	1.27
Information	1.31	1.36	1.56**	1.01	0.67*	0.74	1.47*
LR χ^2^	23.03**	31.26**	36.88**	32.19**	19.49*	23.60**	24.41**

The numbers show the odds ratios. ^*a*^1, male; 2, female. ^*b*^1, single; 2, married. *N* = 210; * *p* < 0.05, ** *p* < 0.01.

We also found that personality traits were significantly correlated with motivations. Hierarchical regressions (Table 6) showed that extraversion was a positive predictor of social motivation (β = 0.29, *p* < 0.01) and openness significantly predicted information motivation (β = 0.28, *p* < 0.01).

**TABLE 6:** Hierarchical regressions predicting motivations with personality traits.

	**Social**	**Entertainment**	**Information**
Openness	0.07	0.00	0.28**
Conscientiousness	–0.13	0.11	0.07
Extraversion	0.29**	0.17	0.05
Agreeableness	–0.15	0.03	–0.12
Neuroticism	–0.09	0.13	–0.11
Adjusted *R*^2^	0.12	0.00	0.16

All demographic variables were controlled for. The numbers show standardized beta coefficients. *N* = 210; * *p* < 0.05, ** *p* < 0.01.

**TABLE 7:** Hierarchical regressions examining viewers’ behaviors with personality traits and motivations.

	**Days**	**Streams**	**Chat**
Social	0.03	0.27**	0.12
Entertainment	0.07	–0.11	–0.00
Information	0.21*	–0.05	0.16
Openness	0.17	0.13	0.23**
Conscientiousness	–0.12	0.09	0.00
Extraversion	–0.06	−0.24**	–0.09
Agreeableness	–0.04	0.00	–0.05
Neuroticism	0.02	0.08	0.07
Adjusted *R*^2^	0.08	0.10	0.15

All demographic variables were controlled for. The numbers show standardized beta coefficients. *N* = 210; * *p* < 0.05, ** *p* < 0.01.

**FIGURE 1:**
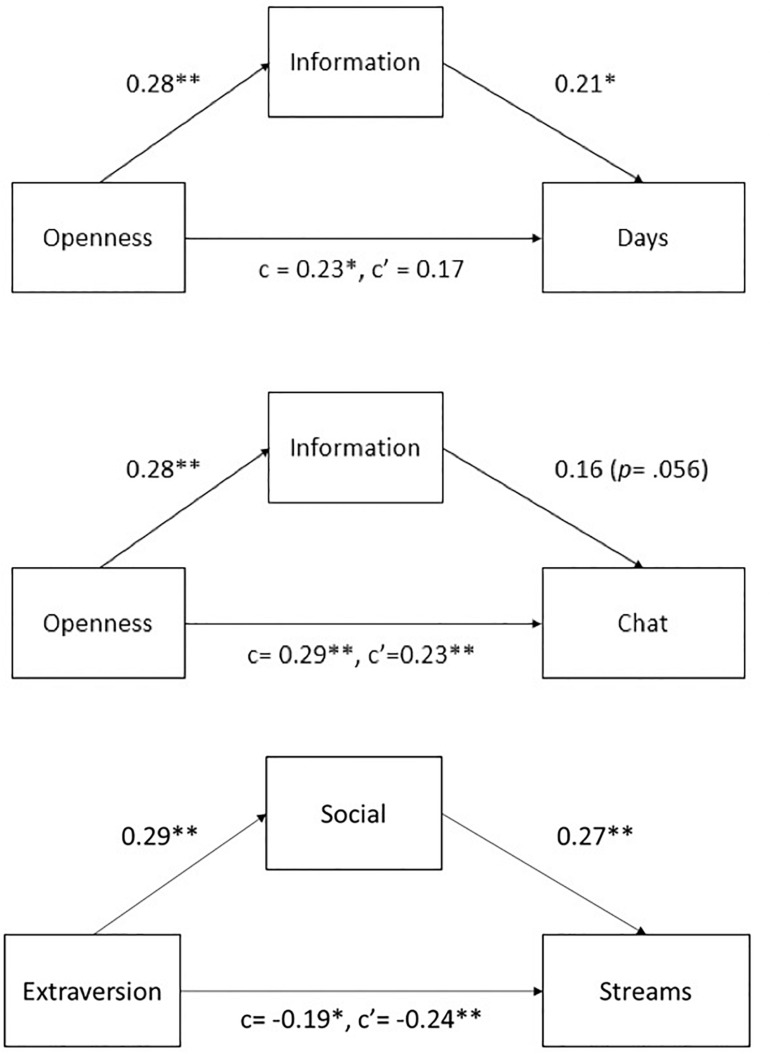
Mediation of motivations between personality traits and viewers’ behaviors. The numbers show standardized beta coefficients: *N* = 210; ^∗^*p* < 0.05, ^∗∗^*p* < 0.01.

To explore the mediation relationship between personality traits and viewers’ live streaming use, we conducted a regression analysis with the motivations controlled for Table 7. We found that information mediated the effect of openness (Figure 1). After controlling for the effect of information, openness was no longer a significant predictor of days [β = *0.23, p* < *0.05*; mediated β′ = *0.17*, *p* = *0.069*; Sobel test *z* = 1.97, *p* < 0.05, variance accounted for (VAF) = 22.6%]. The coefficient of chat became smaller (β = 0.29, *p* < 0.01; mediated β′ = 0.23, *p* < *0.01*; Sobel test *z* = 1.72, *p* = 0.08, VAF = 14.5%); however, the mediation on chat was not significant. Interestingly, we also found an inconsistent mediation from extraversion to streams through social motivation (MacKinnon et al., 2007). After controlling for the effect of extraversion, social was a positive predictor of streams (β = 0.27, *p* < 0.01). This shows that the use of live streaming by extraverts is motivated by social needs. However, after controlling for the effect of social, extraversion had an even stronger negative relation with streams (β = −0.19, *p* < *0.05*; mediated β′ = −0.24, *p* < *0.01*, Sobel test *z* = 2.11, *p* < 0.05, VAF = −36.6%). Here social motivation acted as a suppressor variable. Among participants who were extraverted, those who were more socially motivated tended to use live streaming less. These evidences support our H7a and H7c that motivations mediate the effects of personality traits in live streaming use.

Finally, logistic regressions were conducted to analyze tipping behavior. First, we analyzed tipping behavior with viewing and chatting behavior. The results showed that people who chatted more (OR = 2.02, *p* < 0.01) were more likely to spend money on tipping. However, whether to tip was not associated with viewers’ demographics (*p* ≥ 0.07). Then, we examined tipping behavior with motivations. The results showed that people who were motivated by social (OR = 2.25, *p* < 0.01) were more likely to spend money on tipping. These results support H8 that social interaction is positively associated with tipping behavior.

## Discussion

The current study tried to answer the question based on an analysis of Chinese viewers: How do individual differences in terms of personality traits and motivations affect live streaming use? In particular, how does the mediation relationship between personality traits and motivations can benefit our understanding? Furthermore, we discuss how culture may influence tipping behavior in live streaming.

### How Do Personality Traits and Motivations Affect Live Streaming Use

The findings provide a personality profile of Chinese live streaming users as more open and less extravert. This demonstrates that live streaming is different from SNSs like Facebook that affords self-presentation to gain popularity among others. In such platform, the extrovert users engage more actively by status updating or commenting, while neurotic users are more likely to post contents and gain social support (Shen et al., 2015). The community of live streaming is centered on the interaction of creating and sharing content of diverging interests. Live streaming in China accommodates broad topics, such as education or creative project making; it can fit the diverse interests of open people (Lu et al., 2018, 2019a). This attracts high openness personality profiles of viewers. Among all the personality traits, openness was a strong predictor of live streaming use in terms of both days per week of watching and chat frequency. The content of live streaming also supports the community. In our study, neuroticism was not associated with any live streaming use. Our hypothesis that neuroticism was associated with more live streaming use was built on the proposition that neurotic people use new media to seek support and companionship. This is supported by the positive relationship between neuroticism and use of chat rooms (Hamburger and Ben-Artzi, 2000) and instant messaging (Ehrenberg et al., 2008). However, the viewers of live streaming are rather atomized as their comments are more often around the streamers and broadcasting. Viewers have less chance to build relationships with others and find group identification. It is also reflected in our results that the association between neuroticism and social motivation was not significant. This may explain why our hypothesis was not supported.

In general, personality traits have less influence on genre selection than motivations. Our results showed that entertainment was associated with preference *of games and performance* streams. Moreover, different genres attract viewers with different motivations. For example, performance streams attract viewers who seek social interaction and entertainment but not those who look for information. Education streams were likewise less chosen by viewers motivated by entertainment. It seems that entertainment was more related to genre selection than general usage frequency, whereas information was strongly associated with usage frequency and communication behavior. Among the three motivations, social interaction not only predicted genre selection (games, performance, and celebrity) but also viewing frequency and tipping behavior. According to Gros et al. (2017), socialization was correlated with time and money spent on Twitch, while entertainment and time spent exhibited a low degree of correlation. It seems that social interaction, information, and entertainment could motivate viewers to participate in live streaming, while the strength of their influence and the aspects of user behavior (e.g., watch frequency, chat, and genres) they affect can be varied. Future research can explore this further in consideration of platform differences and other measurements of user behavior.

In addition, we explained how motivations can mediate the effects of personality traits to influence user behaviors in live streaming. Dweck and Leggett (1988) presented a model for motivational and personality processes and demonstrated that both situational variables and dispositional variables play important roles in producing behavior. Many previous studies examined social media use by either personality traits or by motivations. We suggest that person–situation interaction could be a better way to understand user behavior. For example, the inconsistent indirect effect showed that social motivation suppressed extraverts’ likelihood of participating in live streaming, which explained the negative association between extraverts and live streaming use. It is possible that extraverts cannot promote themselves freely and carry out their social skills as viewers in live streaming. Our results also indicated that information mediated the relationship between openness and live streaming use. As Chinese live streaming platforms provide viewers with free access of all content, it could facilitate the need of information seeking and well accommodate people high in openness. Therefore, the interpretation of both personality traits and motivations can yield valuable insight into live streaming use.

### How Culture May Influence Tipping Behavior in Live Streaming

One feature of live streaming is that users can directly provide financial support to streamers. According to survey, among 31.5% of users who spent money on Twitch, majority paid for subscription (Gros et al., 2017), whereas in China, 40% of users paid for streams all through virtual gifting and, remarkably, 5% of users spent more than 134 USD (equal to 1,000 RMB) monthly. Culture may influence the tipping behavior in live streaming. Evidence suggests that the cultural orientation of vertical collectivism can predict buying virtual goods with real money in social network gaming (Lee and Wohn, 2012). Triandis (1995) demonstrated two types of collectivism: horizontal collectivism, which emphasizes group membership and equality among group members, and vertical collectivism, which indicate the difference in status and hierarchy among group members. On the one hand, our results indicated that people are more likely to spend when they engage with others. Zhu et al. (2017) analyzed the tipping data from *Douyu* platform and found that viewers often follow others to send virtual gifts. If virtual gifting is the social norm in Chinese live streaming, viewers who want to find group identity need to adopt this practice. In this sense, virtual gifting could be a conformity behavior motivated by the group identity. On the other hand, in many Chinese live streaming platforms, there are a number of gift types with a wide range of values, from a 0.1 RMB “like” to a 500 RMB “rocket”. Sending high-value gifts to streamers can create special visual or audio effects, such as flashing lights through the browser window, and can attract attention from streamers and other viewers. The platforms also designed different badges and titles to distinguish viewers who send high-value gifts from other viewers who paid less and provided those high-value gift senders with privileges. These symbolically demonstrated that hierarchies attract people who seek status in live streaming and motivate them to enhance their presence through tipping behavior (Li et al., 2018). On *Douyu* platform, the 2.7% high-value gift senders contributed to 80.2% of the total gift value (Zhu et al., 2017). It seems that both horizontal collectivism and vertical collectivism could contribute to the phenomenon of virtual gifting in China, which is reflected by the different dynamics of social interaction. Future research can investigate this question with more refined measurement on tipping behavior through a cross-cultural analysis.

### Limitations, Future Research, and Implications

Our current research provides a personality profile of Chinese live streaming users and corroborates previous evidence on the motivations of live streaming use. Importantly, our findings contribute novel insight into how person–situation jointly could be used in interpreting viewers’ livestreaming use. Theoretically, this expands the perspective on individual difference in livestreaming use. Viewers with different personality traits could be motivated by different needs, which together shape their livestreaming use.

Practically, the findings of the current study suggest that strategies targeted to different viewer’s personality profile can be applied to attract users. For neurotics who cannot find enough social support in livestreaming, platforms could promote small-sized channels where a close interaction is allowed. Platforms could support economic and inclusion opportunities for streamers with disabilities, mental health issues, or physical health issues. These streamers can build up an inclusive community for people who encounter similar difficulties in life through sharing experiences and encouraging each other, which can be beneficial to both streamers and viewers (Johnson, 2019). For extravert viewers who need more exposure and cannot be satisfied with current infrastructure in live streaming, platforms can design functions to recognize viewers who contribute to the channel by posting, commenting, and participating other than virtual gifting with additional prizes. Viewers can attract followers, which can maintain their enthusiasm and extend their social network. Other functions to facilitate direct interaction between viewers can also enhance the interaction between viewers and develop a sense of group identity.

Although this research offers new insight into the individual difference of livestream viewers, it has some limitations. First, viewers’ behavior can be defined in different ways. Besides the variables that we used, duration of streams watching and interaction behaviors can also describe different aspects of live streaming use. Regarding the tipping behavior, although we did not find an association between income level of viewers and whether they chose to spend, the findings could have been restricted by the measure. Determining how much users pay for tipping or how often they tip could provide more details to explain this behavior. Second, our study surveyed users across platforms and explored the general usage patterns of live streaming as a homogenous activity. However, platform and content differences were not included, which could be a noteworthy factor in determining user behaviors (Lu et al., 2019a). Although most viewers select multiple genres, user behaviors and motivation can be varied. In addition, viewers watch live streams not only *via* SSLSs but also by some other applications. For example, *Taobao*, the biggest online shopping platform, used live streaming for product sales. The relationship between viewers’ behaviors and personality traits and motivations can change as the context changes. Further research could examine specific platforms, content, and new application to understand the use of live streaming.

## Conclusion

The current study is one of initial attempts to explore viewers’ personality traits and motivations related to live streaming use in the general population in China. Results indicated that extraversion and openness were two major personality traits, and social, information, and entertainment were the three motivations that associated with viewers’ live streaming use. Furthermore, we found that motivations can mediate the effects of personality traits, demonstrating that personality traits and motivations jointly influence live streaming use. It provides novel insight into understanding the various degrees of social interaction in live streaming from individual differences. The personality profile of Chinese viewers can also support a future cross-cultural study to understand live streaming user behaviors.

## Data Availability Statement

The datasets generated for this study are available on request to the corresponding author.

## Ethics Statement

The studies involving human participants were reviewed and approved by USC-SJTU Institute of Cultural and Creative Industry Ethnic Board. The participants provide their written informed consent to participate in this study.

## Author Contributions

YY was responsible for collecting data and part of the analysis. YX was responsible for part of the analysis and all other work including idea generation, analysis and writing. All authors contributed to the article and approved the submitted version.

## Conflict of Interest

The authors declare that the research was conducted in the absence of any commercial or financial relationships that could be construed as a potential conflict of interest.

## Figures and Tables

**TABLE A1:** Factor analysis of motivations.

**Items**	**Factor 1 social**	**Factor 2 entertainment**	**Factor 3 information**
1. Find people with same interests	0.71		
2. Develop close relationship	0.74		
3. Maintain current relationship	0.76		
4. Find companion	0.69		
5. To communicate with others	0.78		
6. Develop business relationship	0.69		
7. Support talented streamers	0.69		
8. Follow the trend	0.49		
9. Relieve boredom		0.51	
10. Cheer myself up		0.69	
11. Feel relaxed		0.67	
12. Forget my problems		0.49	
13. To pass the time		0.67	
14. Get new information			−0.58
15. Learn or improve skills			−0.33
Proportion, %	32.15	16.31	10.53
Cumulative, %	32.15	48.46	58.99
			

**TABLE A2:** Zero-Order Correlational Matrix of Personality Traits, Motivations, and Live Streaming Use.

**Variables**	**1**	**2**	**3**	**4**	**5**	**6**	**7**	**8**	**9**	**10**
1. Openness										
2. Conscientiousness	0.43**									
3. Extraversion	0.60**	0.41**								
4. Agreeableness	0.32**	0.44**	0.13*							
5. Neuroticism	−0.27**	−0.45**	−0.38**	−0.44**						
6. Social	0.20**	0.01	0.30**	–0.08	–0.10					
7. Entertainment	0.10	0.11	0.16*	0.04	0.01	0.14*				
8. Information	0.36**	0.24**	0.31**	0.08	−0.20**	0.56**	0.04			
9. Days	0.19**	–0.02	0.08	–0.03	0.01	0.20**	0.07	0.25**		
10. Lives	0.07	0.02	–0.07	–0.00	0.04	0.21**	–0.09	0.11	0.26**	
11. Chat	0.27**	0.06	0.14*	–0.00	0.01	0.27**	0.03	0.32**	0.43**	0.23**

*N* = 210; * *p* < 0.05, ** *p* < 0.01.

## Footnotes

1 www.sojump.com

2 The participants were asked to indicate the number of streams that they watch per day regardless of the length of each stream. The duration of a stream can be controlled by each streamer, while some streams may last from 1 to 3 h.

3 iResearch is a leading provider of online audience measurement and consumer insights in China. The company has provided research reports on live streaming since 2015. They published the 2016 China’s mobile video streaming viewers report based on a survey of 1,265 users.

## Appendix 1. Motivations in Live Streaming Use

Participants were recruited through referral (snow-ball sampling). They have used different live streaming platforms such as *Douyu*, *YY*, *Bilibili*, *Momo*, *Huya*, *etc*. They were asked questions about their use experience of live streaming to assure that they are active users. Then, they were asked to provide ideas about motivations of live streaming using elaboration *via* online chat (approximately 10 to 30 min), depending on the details that were provided within the open-ended questions.

The chat transcript was analyzed by a conventional approach (Hsieh and Shannon, 2005). The coded items identified more than two times were selected. These items were compared with items from existing scales. Then, the items were adjusted and rephrased to fit in the live streaming context. For example, “maintain and improve relationship” (Lee and Wohn, 2012) was adjusted to “develop close relationship” and “develop business relationship”. The final 15 items in the scale were discussed by a research team who met to gain consensus.
